# First isolation and characterization of caprine oviduct fluid extracellular vesicles

**DOI:** 10.1590/1984-3143-AR2024-0039

**Published:** 2024-10-21

**Authors:** Roberto Mendes, Agostinho Soares de Alcântara, Gildas Mbemya Tetaping, Marco Aurélio Schiavo Novaes, Vanessa Barbosa Pinheiro Gonçalves, João Xavier da Silva, José Jonathas Albuquerque de Almeida, Maria Izabel Florindo Guedes, Luzia Kalyne Almeida Moreira Leal, Joanna Maria Gonçalves Souza-Fabjan, Roberto Nicolete, Deborah de Melo Magalhães Padilha, José Ricardo de Figueiredo, Ana Paula Ribeiro Rodrigues

**Affiliations:** 1 Laboratório de Manipulação de Oócitos e Folículos Ovarianos Pré-antrais – LAMOFOPA, Faculdade de Medicina Veterinária, Universidade Estadual do Ceará – UECE, Fortaleza, CE, Brasil; 2 Faculdade de Educação e Ciências Integradas de Crateús, Universidade Estadual do Ceará – UECE, Fortaleza, CE, Brasil; 3 Faculdade de Veterinária, Universidade Federal Fluminense – UFF, Niterói, RJ, Brasil; 4 Fundação Oswaldo Cruz – Fiocruz Ceará, Eusébio, CE, Brasil; 5 Laboratório de Biotecnologia e Biologia Molecular, Centro de Ciências da Saúde – CCS, Universidade Estadual do Ceará – UECE, Fortaleza, CE, Brasil; 6 Departamento de Farmácia, Faculdade de Farmácia, Odontologia e Enfermagem, Universidade Federal do Ceará – UFC, Fortaleza, CE, Brasil; 7 Rede Nordeste de Biotecnologia, Programa de Pós-graduação em Biotecnologia, Universidade Estadual do Ceará – UECE, Fortaleza, CE, Brasil; 8 Programa de Pós-graduação em Biotecnologia, Universidade Potiguar, Natal, RN, Brasil

**Keywords:** estrous cycle, nanoparticles, oviductosomes, physicochemical characteristics

## Abstract

Oviduct fluid extracellular vesicles (oEV) are essential for periconceptional events. The presence of EV has already been identified in the oviduct fluid (OF) from mammalian species, except in caprine. Therefore, this study aimed to isolate and characterize the caprine oEV (coEV). Initially, in Experiment 1, coEV were isolated from the OF of either each animal individually or from a pool of three animals. In experiment 2, coEV were isolated during the follicular or luteal phases of the estrous cycle. The coEV were characterized by size distribution, polydispersity index (PDI), and zeta potential using dynamic light scattering (DLS) analysis, as well as, by transmission electron microscopy (TEM) and dot blotting (DB). Our results indicated that the physicochemical characteristics of the coEV were similar (P > 0.05), regardless of the isolation method (individual or pool). However, coEV collected during the luteal phase were larger (P < 0.05) than those during the follicular phase. The TEM showed spherical and cup-shaped particles, characteristic of exosomes. The DB revealed the presence of exosomal proteins involved in the biogenesis of coEV. In conclusion, it is possible to isolate and characterize coEV from a single caprine female and the estrous cycle phase influences the vesicles average size and PDI.

## Introduction

Oviduct fluid extracellular vesicles (oEV) are nanoparticles formed by the cell plasma membrane phospholipid bilayer. They are classified into large EV, including microvesicles (>200 nm), which are formed by the evagination of the plasma membrane, and small EV such as exosomes (< 150nm-200nm), with endocytic origin released via fusion of multivesicular bodies with the plasma membrane. The EV found in biological fluids facilitates the transport of lipids, proteins, and nucleic acids, by intercellular communication (Alcântara-Neto et al., 2020).

According to Almiñana and Bauersachs (2019), oEV are a tool capable of maximize the efficiency of assisted reproductive techniques (ARTs) by mimicking the oviduct microenvironment in livestock species. Generally, oEV isolation is performed from a pool of animals to obtain more samples. However, isolation from a pool leads to the loss of individual EV characteristics, masking the vesicular profile of each animal. Therefore, isolating oEV from a single female, allows to identify the best suitable animals for biotechnological applications (Lennon et al., 2019).

The production and morphomolecular profiles of oEV are regulated by steroid hormones, which modulate reproductive events such as sperm capacitation, fertilization, and embryogenesis. These steroid hormone levels impact the production of bovine oEV, demonstrating the influence of the estrous cycle on EV (Almiñana et al., 2018). However, studies with isolation and characterization of oEV in some economically important species, such as goats, remain non-existent.

Considering the relevance of oEV to periconceptional events and ARTs optimization, and the lack of reports in goats, this study aims to isolate and characterize oEV obtained from individual animals or pooled samples during the follicular and luteal phases of the estrous cycle.

## Methods

### Ethical approval

This study was conducted in accordance with the Animal Management and Ethical Regulation Committee recommendations of the State University of Ceará (01095870.2022).

### Chemicals

Unless otherwise stated, the reagents and chemicals used in this study were manufactured by Sigma-Aldrich Chemical Co. (St. Louis, MO, USA). The HSPA1A (ab2787) and CD63 (ab68418) antibodies were manufactured by Abcam (Inc. Abcam, Cambridge, MA, USA), and CYP17A1 (bs-6695R) were manufactured by Bioss (Bioss Inc., Woburn, MA, USA).

### Experimental design

This study was divided into two experiments. Experiment 1 was performed to isolate and characterize oEV isolated individually or using oviduct fluid (OF) pool from three animals. Then, 17 oviduct pairs from crossbred goats were used: eight were processed individually, and nine were processed in pool (three pairs per pool) to obtain oEV. In Experiment 2, 21 pairs of oviducts were used, 10 obtained from follicular phase and 11 from luteal phase of the estrous cycle. The identification of the follicular or luteal phases was performed according to the goat ovary morphology (Kandiel et al., 2010). The follicular phase was characterized by the presence of at least four antral ovarian follicles (3-8 mm) with an antrum filled with translucent slightly reddish fluid in the cortical region of the ovary. The luteal phase, on the other hand, was characterized by the presence of a rosy corpus luteum with a diameter ≥ 6 mm.

### Extracellular vesicles isolation

The caprine oviducts were obtained in a local slaughterhouse and dry-transported to the laboratory at 4 °C. Each oviduct lumen was flushed with a 1 mL of phosphate-buffered saline (PBS) and gently pressed from the ampulla to the isthmus. The fluids from both oviducts of the same females were collected and centrifuged: 300 xg, and at 12,000 xg both at 15 min, 4 °C. After the second centrifugation the final volume of supernatant (clarified OF) was stored at –80 °C. The OF from each female or from a pool of three females were ultracentrifuged at 100,000 xg for 90 min at 4 °C, using a swing rotor (SW 28.1; 14.2; Quick-Seal®; 356291). The supernatant (EV-depleted OF; DOF) was collected, the pellet was resuspended in 14 mL of PBS and ultracentrifuged again under the same conditions. The final pellet was resuspended in 150 µL of PBS and stored at –80 °C until the EV characterization.

### Dynamic light scattering (DLS) analysis

The size distribution, polydispersity index (PDI), and zeta potential (ZP) of coEV were measured by DLS analysis using the Zetasizer Nano ZS. Individual or pooled isolated oEV samples were were diluted in PBS (1:50 for individual samples; 1:200 for pooled samples) to a final volume of 1 mL and analysed according to the manufacturer's recommendations.

### Transmission electron microscopy (TEM)

The ultrastructure of coEV was characterized by Transmission electron microscopy (TEM). The coEV aliquots were fixed with TRUMP solution. Sample (3 µL) was placed on a formvar-carbon-coated grid, treated with 0.1% poly-L-lysine for 5 min. The grids were counterstained with 2% uranyl acetate for 2 min, washed with ultrapure water, and air-dried at 37 °C. Micrographs were obtained using a JEM 1011 TEM (JEOL, Japan) at 80 Kv.

### Dot blotting (DB)

The protein concentration in OF, DOF and EV samples were quantified as described by Bradford (1976). Aliquots of 2 µg/µL of OF, DOF, and EV samples from females in follicular (n=5) or luteal (n=5) phase were applied to nitrocellulose membrane, air-dried, and blocked with Tris-buffered saline with Tween 20 (0.05%; PBST) supplemented with lyophilized low-fat milk (1% w/v) for 60 min. Afterwards, three membranes with the same samples were washed with PBST and incubated with the primary antibodies rabbit anti-CD63 (ab68418; 1:1000), mouse anti-HSPA1A (ab2787; 1:5000), and mouse anti-CYP17A1 (bs-6695R; 1:5000) for 60 min at 37 °C. Then, the membranes were washed with PBST and incubated with the secondary antibodies HRP-conjugated anti-rabbit IgG (A6154; 1:5000) and anti-mouse IgG (G21040; 1:5000). Then, both membranes were washed with PBST, and revealed Clarity™ Western ECL Substrate. Membranes without first antibodies, PBS and water samples were used as negative controls.

### Statistical Analysis

The data were described as mean ± standard error of the mean (SEM). Data from both experiments were analyzed by Shapiro-Wilk normality and compared by the Mann-Whitney tests, using the Statistical Package for Social Science (SPSS - version 23.0) software. The outliers were excluded by the Z-test, and differences were considered significant when P<0.05.

## Results

**Figure 1:**
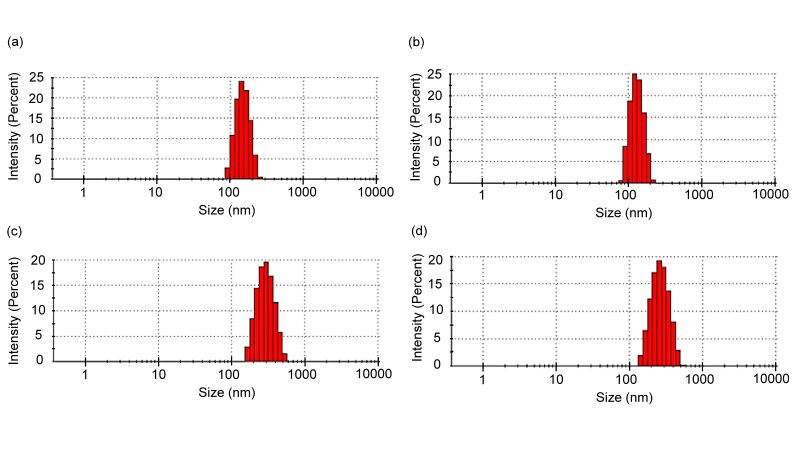
Size distribution of caprine oviductal extracellular vesicles (coEV). Representative images from Dynamic Light Scattering analysis showing the size distribution of oEV isolated individually (a) and from a pool of three animals (b), or individually during the follicular (c) and luteal (d) phases of the estrous cycle.

The coEV characterization by size, PDI, and ZP is shown (Table 1). No statistical differences were observed in any parameters when comparing individual and pooled groups. Nevertheless, the average size of the oEV collected during the follicular phase was significantly lower than oEV during the luteal phase. Similar results were observed for the PDI, where the polydispersity of follicular group was lower than luteal group. However, no significant differences were observed for the ZP between both phases. The Figure 1 shows the representation of size distribution from each experimental group.

**Table 1:** Mean size (nm), polydispersity index (PDI), and zeta potential (mV) of caprine oviductal fluid extracellular vesicles (coEV).

**Experiment**	**Group**	**Particle size (nm)**	**Polydispersity (PDI)**	**Zeta potential (mV)**
**Experiment 1**	Individual	262.7 ± 14.3^a^	0.33 ± 0.02^a^	-14.7 ± 0.67^a^
	Pool	269.1 ± 17.5^a^	0.30 ± 0.04^a^	-13.3 ± 0.77^a^
**Experiment 2**	Follicular phase	274.8 ± 12.1^a^	0.29 ± 0.01^a^	-13.2 ± 0.26^a^
	Luteal phase	345.4 ± 14.5^b^	0.39 ± 0.02^b^	-12.4 ± 0.49^a^

^a,b^Different letters within the same experiments indicate significant differences by Mann-Whitney Test (P <0.05).

**Figure 2:**
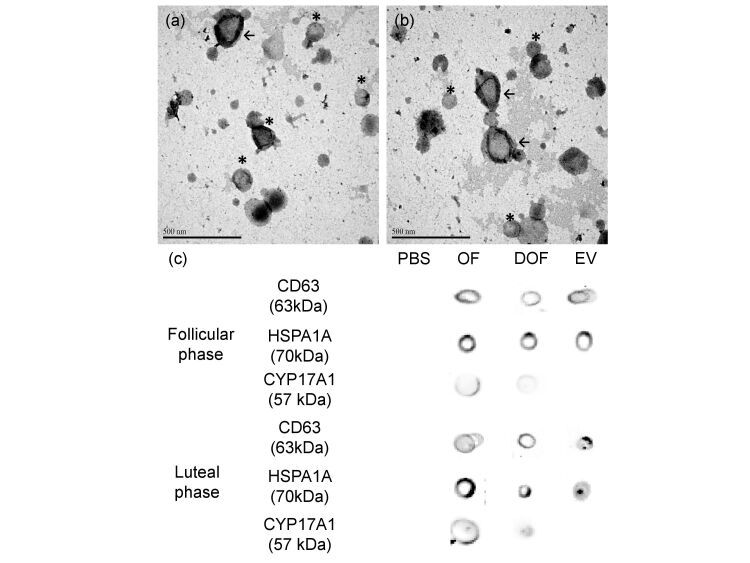
Morphological and molecular characterization of caprine oviductal fluid extracellular vesicles (coEV). (a), (b): Representative ultrastructure images of microvesicles (arrow; >150 nm diameter) and exosomes (asterisks; 50-150 nm diameter) identified in coEV samples through TEM; (c) Immunodetection of known exosomal protein markers (CD63 and HSPA1A) in oviductal fluid (OF), oviductal fluid depleted of vesicles (DOF), extracellular vesicles (EV) samples, and EV negative protein marker (CYP17A1).

The TEM confirmed the presence of nanoparticles with a diameter of 50-350 nm, classically associated with coEV. The images allowed the identification of two coEV types: microvesicles with 150-350 nm and exosomes with 50-150 nm in “cup shape” (Figure 2a and b). The total protein concentration in coEV samples was 1.0 µg/µL for follicular phase and 0.8 µg/µL for luteal phase. The DB confirmed the presence of exosomal proteins CD63 and HSPA1A in the coEV, and goats OF. Besides, the coEV samples were negatives for CYP17A1, indicating absence of cell contamination in coEV (Figure 2c).

## Discussion

This is the first study related to the isolation and characterization of coEV. Based on this information and the absence of caprine reports, we evaluated the coEV physicochemical characteristics, which were similar between the coEV isolated from individual or pool group. However, coEV in follicular phase had a size significantly smaller compared to those in the luteal phase. This differs in murine (Yi et al., 2022) and swine (Laezer et al., 2020), whose oEV in follicular phase were larger than in the luteal phase. However, in bovine no difference in the size distribution of oEV were observed during estrous cycle. These variations on coEV size according to estrous cycle suggest species-specific influences due to physiological differences (Almiñana et al., 2018).

The PDI of our coEV indicate a heterogeneous size distribution. The PDI reflects the homogeneity or heterogeneity of EV size distribution. Lower PDI value (closer to zero), suggest more homogeneity to nanoparticles, while higher value (closer to one), suggest a more heterogeneity sample. Additionally, PDI values between 0.05 and 0.7 are the most appropriate for DLS analysis, as values below or above this range are unreliable (Danaei et al., 2018).

The coEV had a negative surface charge, similar to found in biological nanoparticles due to molecular content similar to present in the cells, contributing to their negative charge (Jamaludin et al., 2019). According to Wright et al. (2022) the ZP is affected by salts, detergents, and the buffer type used for resuspension. These authors used DPBS for EV resuspension. Here, we used PBS, which is rich in salts, and our ZP were similar to them. Therefore, ZP are related to the presence of molecules and crucial for evaluating the interaction of EV with cells and understanding their activity (Bunggulawa et al., 2018).

Our results of CD63 and HSPA1A corroborating with Huang et al. (2017) and Alcântara-Neto et al. (2020), who also reported the presence of CD63 and HSPA1A in oEV. These proteins found in EV plays an important role in reproductive events (Almiñana et al., 2018). Moreover, we observed the absence of CYP17A1 in our coEV samples. Corroborating with Javadi et al. (2022), which also observed the same result for mitochondrial protein cytochrome C (CYCS), indicating the absence of cell contamination in EVs samples.

## Conclusion

In conclusion, it is possible to isolate and characterize individually coEV, and that the estrous cycle phases influence their mean size and PDI. Considering the role of oEV, this study represents the first step coEV isolation and characterization, which can be used as a base protocol for further studies in goat, such as reproductive markers or media supplementation to improve ARTs.
